# New Chemically Resistant Coating Systems with Progressive Incorporation of Hazardous Waste in Polyurethane and Epoxy Matrices

**DOI:** 10.3390/ma15093235

**Published:** 2022-04-29

**Authors:** Jakub Hodul, Lenka Mészárosová, Rostislav Drochytka

**Affiliations:** Faculty of Civil Engineering, Institute of Technology of Building Materials and Components, Brno University of Technology, Veveri 95, 602 00 Brno, Czech Republic; meszarosova.l@fce.vutbr.cz (L.M.); drochytka.r@fce.vutbr.cz (R.D.)

**Keywords:** epoxy resin, polyurethane resin, coating, hazardous waste, filler, chemical resistance, concrete protection

## Abstract

New types of highly chemically resistant coating systems, primarily intended for concrete and metal substrates, were designed and experimentally verified in the paper. Secondary raw materials in optimal amounts, including solidified hazardous waste (e.g., end product and cement bypass dust), were used as microfillers. The polymer coating systems, containing pre-treated hazardous waste (HW), showed high abrasion resistance and excellent adhesion to metal and concrete surfaces. Based on polyurethane and epoxy resins, the coatings can be used in environments where aggressive chemical media act, such as sewers and the chemical industry. The developed polymeric coating systems showed even better properties than the compared reference coating systems. The chemical resistance of the three-layer coating systems was evaluated both visually and based on changes in mechanical properties, such as hardness and adhesion. The microstructure of the coating systems was also monitored using a digital optical microscope and a scanning electron microscope with energy dispersive X-ray analysis (SEM-EDX) after chemical stress. It was observed that the particles of HW were fully incorporated into the polymer matrix of the coating systems.

## 1. Introduction

Coating systems are designed based on their intended use. The primary requirements for polymer coating systems are their resistance to aggressive environments as well as their durability [1,2]. In aggressive environmental conditions where the structures are exposed to degradations, providing some protective layers can effectively increase the service life of the structures [3,4]. The degradation of polymeric coating materials is controlled by environmental factors, such as light, oxygen, moisture, salt, acids, temperature, cyclic loading, etc. [5]. There is a wide range of basic bases for coating materials. Polymer materials produced by polyaddition, mainly on polyurethane and epoxide bases, are effective in an aggressive environment.

Epoxies are known for their excellent adhesion, chemical and heat resistance, good-to-excellent mechanical properties and very good electrical insulating properties [6,7]. These are compounds containing more than one epoxide (oxirane) group, which are highly reactive and create cross-linked macromolecular products in one molecule. Commercial epoxy resins that contain aliphatic, cycloaliphatic or aromatic backbones are available in a wide range of molecular weights, from several hundreds to tens of thousands [8]. It is well known that coatings with high resin molecular weights are superior in barrier performance, because more interdiffusion of polymer chains occurs between large resin molecules, and lead to a better cross-linking property [9]. Epoxy resins (ER) have been widely used and studied as anti-corrosion coatings [10] and highly resistant materials because of their excellent mechanical properties and chemical stability [11]. Epoxy coatings provide adequate protection to concrete [4,12]. It has been reported that the addition of fillers, including particles and fibres, can improve their strength, modulus and impact toughness [13]. The inclusion of optimum amounts of fillers, pigments, additives and nanomaterials in the epoxy coating matrix could remarkably increase its corrosion resistance [14,15]. Although waterborne epoxy coatings have been commercialised for more than 40 years, they still share only a small percentage of the total market among anticorrosive coatings [16]. The demand for environmentally friendly water-based coating systems has increased continuously over the last decades, as they become more important due their low VOCs emission than solvent based coatings [17]. Waterborne epoxy/amine coatings, compared with solvent based, show considerably lower chemical resistance [18]. Strong adhesion also prevents moisture vapor from passing through the coating and condensing in a poor area of adhesion, which may lead to the blistering of the coating [19]. Many efforts have been made to improve the anticorrosive property of waterborne coatings [20,21].

Polyurethane is a synthetic thermosetting polymer that can be used to provide structural enhancement, an optimum indoor temperature and protection against the environment [22]. Polyurethanes are usually obtained by the addition polymerisation of polyisocyanates with polyhydric alcohols. However, as Boisaubert showed in his work [23], it is possible to prepare polyurethanes without isocyanates by photocrosslinking. UV-curing coatings usually involves intermolecular interactions such as intramolecular chemical bonding after curing, which lead to the inevitable shrinkage of the cured coatings [24]. Ester linkage provides flexibility, resistance to chemicals, high thermal stability and low flammability to the polymers [25]. Polyurethane coatings can be used on metal substrates [26], and they are used to protect the substrate material from long-term cavitation erosion damage [27]. Despite the abovementioned advantages, compared with solvent-based polyurethanes, waterborne polyurethanes also have some problems, such as poor mechanical properties and weak water resistance [28,29].

When preparing polymer composites, it is advisable to use secondary raw materials as fillers. Their use not only reduces production costs but also the share of polymer fillers. At the same time, using waste that would otherwise end up in a landfill saves primary raw materials, which is very beneficial to the environment. Some authors have investigated the use of waste products in epoxy coatings, such as waste sand in quantities up to 29% by weight [30], jute fibre, abaca fibre and tire particles [31], waste glass fibre [32], bamboo and metal chips [33], pit waste [34], eggshell, wood sawdust and vermiculite [35], fly ash cenospheres [36], electric arc furnace slag [37] and waste glass [38]. It is also possible to use waste tires [39], rice plant waste [40] or fluidised bed combustion fly ash [41] in combination with polyurethane binders.

The problem of using treated hazardous waste (HW) in coating systems has not been solved yet, so this is a new, progressive approach to the use of specific HW. With the suitable treatment of HW, it is possible to develop new types of microfillers for polymer coatings, which can possess the same or even better properties than coatings that use only primary raw materials. Special types of HW, such as cement bypass dust and end product, which are dry powder waste products, are very suitable as the fillers for polymeric coatings. Used HW show mainly toxicity, irritation, and contain heavy metals. By incorporation these types of HW into the primer and middle layer of the polymeric coating systems in the form of solidification products, pollutants will be fixed in polymeric matrices and prevents their leaching into the environment even when used in aggressive conditions.

## 2. Materials

Silica flour and solidified fillers were used as fillers. Three types of binders were used as binding components. These were solvent-free epoxy resin (ERD), waterborne epoxy resin (ERW) and polyurethane resin (PUR).

### 2.1. Fillers

**Figure 1:**
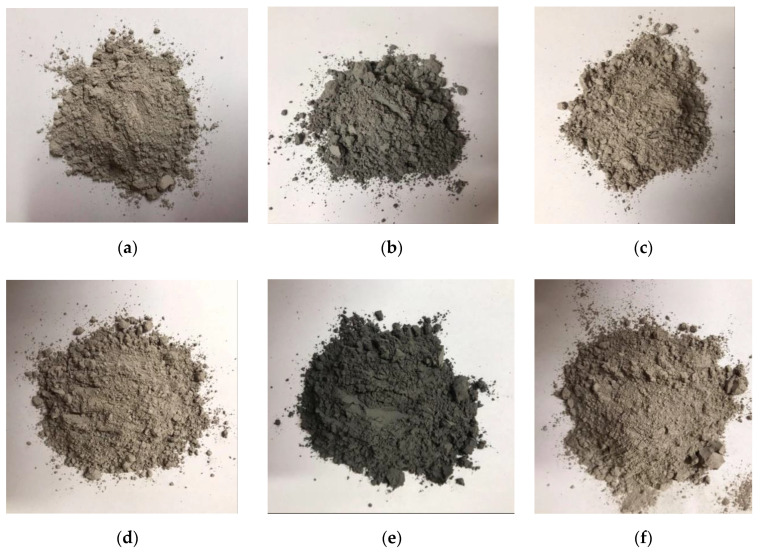
Fillers containing solidification products used in the coating systems: (**a**) SP-P1; (**b**) SP-P2; (**c**) WM-P3; (**d**) SP-E1; (**e**) SP-E2; (**f**) WM-E3.

**Figure 2:**
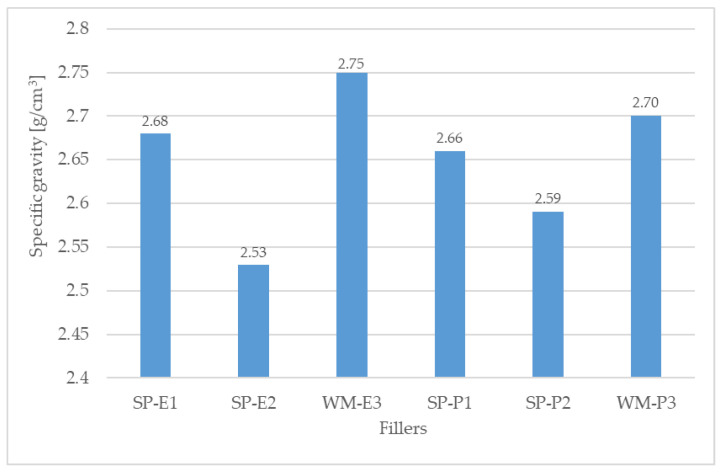
Specific gravity of the fillers.

**Figure 3:**
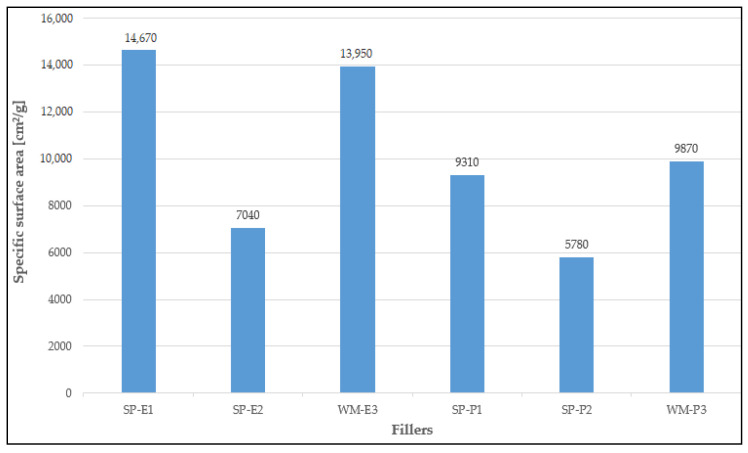
Specific surface area of the fillers.

Silica flour (supplier Chejn, Ltd., Sušice, Czech Republic) was used as a primary filler and also as a filler for the reference coating systems. Six types of solidification products containing different amounts and types of fly ashes and HW were used as alternative fillers (see Table 1). Cement bypass dust, from the cement plant in the Czech Republic, and end product, from the municipal waste incinerator in the Czech Republic, were chosen as the suitable HW. Within the research, two quality levels of the coating systems CS-E and CS-P were tested, while the coating system CS-E contained only a filler composed of HW and fly ash. The solidification products used as microfillers were prepared by first homogenising the input raw materials, then grinding them in a vibratory disc mill to a suitable granulometry. The appearance and structure of individual fillers in the form of solidifiers can be seen in Figure 1a–f. The composition of individual fillers was designed in such a way to ensure the most effective incorporation of pollutants into the polymer matrix, i.e., the first layer of the coating system contained the highest amount of hazardous waste (SP-E1 and SP-P1), which was 10 wt% of cement bypass dust or end product. The filler for the third (sealing) layer of the coating system contained only fly ash and silica flour. For the second layer, fly ash (FF) from high-temperature combustion (class F), as the by-product from the thermal power plant Opatovice, a.s. Czech Republic was used in the filler as a solidification agent, and a filler containing fly ash from fluidised bed combustion (FC), as the by-product from the thermal power plant Kladno, Czech Republic was used for the first and third layers. Both types of fly ashes were by-products from lignite-fired thermal power plants in the Czech Republic; selective non-catalytic reduction (SNCR), which involved the injection of urea into the flue gases for NOx reduction, was used for both of them. Figure 2 shows the specific gravities of the fillers, and Figure 3 shows their specific surface areas. The specific gravity of the filler samples was determined using the AccuPyc II 1340 Pycnometer. It is an automatic gas pycnometer, where the density calculation consists of measuring the volume of helium in a chamber with a sample of known weight. The specific surface area of the fillers was determined using the Blain permeable method in the ZEB MAXAM PC Blain Star instrument. The calculation of the specific surface area is based on the time of air flow through the sample of defined weight, its specific gravity and porosity.

The highest specific weight was determined for the WM-E3 filler, in which only FC was present (see Figure 2). The second highest specific weight was determined for the WM-P3 filler, in which not only FC was used but also silica filler as the primary filler.

As seen in Figure 3, the highest specific surface area was recorded for the SP-E1 filler that contained 90% FC fly ash and 10% cement bypass dust.

**Figure 4:**
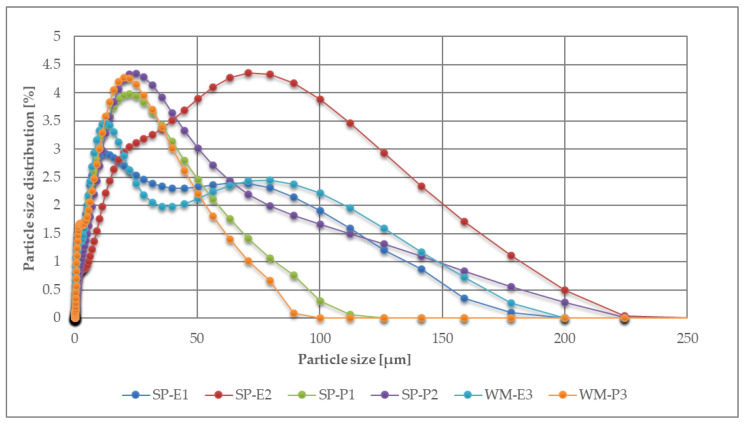
Particle size distribution of the fillers.

The distribution of the particle sizes of fillers used is stated in Figure 4. The particle size of the fillers was measured using the laser diffraction method using the MALVERN Mastersizer 2000, by a dry method. The principle of the measurement is that larger particles reflect laser beams at a smaller angle and with a larger laser intensity, and smaller particles have a larger reflection angle and a lower intensity of the reflected beam. The material is suitable for multiple-layer coating systems if it meets the maximum particle size of 200 μm. This requirement was met by all prepared microfillers. It can be observed that the SP-E2 filler showed, on average, the largest particle size distribution—in the range of 50–200 μm—and thus a smaller specific surface area.

**Figure 5:**
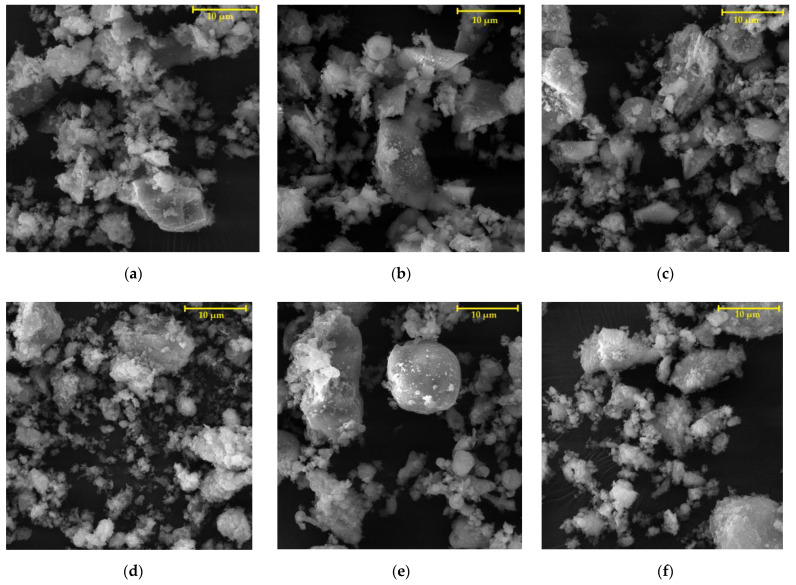
SEM photomicrographs of the fillers, magn. 5000×: (**a**) SP-P1; (**b**) SP-P2; (**c**) WM-P3; (**d**) SP-E1; (**e**) SP-E2; (**f**) WM-E3.

SEM images of the fillers (magnification (magn.) of 5000×) can be observed in Figure 5a–f. Figure 5a–c, which represent fillers (solidification products) for CS-P coating systems, show sharp-edged quartz flour particles, and Figure 5b,e (SP-P2, SP-E2) show cenospheres which contained high-temperature fly ash (FF). Furthermore, it is possible to observe an even distribution of irregularly shaped HW particles between the solidifying agents in the images showing the fillers SP-P1, SP-E1, SP-P2, SP-E2.

### 2.2. Polymer Binders

Three types of polymeric resins were used as binders: solvent-free epoxy resin, water-soluble epoxy resin and polyurethane resin. Manufacturer of these resins is IN-CHEMIE Technology s.r.o. (Olomouc, Czech Republic). The chemical compositions of the polymeric binders used (both components) are stated in Table 2.

### 2.3. Formulations of Coating Systems

The formulations of the coatings that comprised the layers of the coating systems were designed based on the results of the application test, in which the workability, applicability, consistency and quality of the resulting surfaces were evaluated. Only a silica flour filler was added to the coating systems. Generally, more secondary raw materials were used for the CS-E coating system. The formulations of the epoxy coating systems are stated in Table 3 and Table 4, and the compositions of the polyurethane coatings are stated in Table 5.

In general, the thickness of a multilayer coating system ranges between 300 μm and 1 mm. The average thickness of all applied coating systems was 300–400 μm, and this thickness was also met in all reference materials.

## 3. Methods

### 3.1. Dynamic Viscosity

Dynamic viscosity was determined in the fresh coating materials using the Elcometer 2300 viscosimeter (Elcometer Instruments Ltd., Manchester, UK), pursuant to the EN ISO 2884-2 standard [42]. The temperature of each material during viscosity measurement was 23 °C, and the R4 spindle was used for all tested coatings. The optimal speed of the spindle was 100 rpm for all formulations except the coatings that contained 30% WM3 filler.

### 3.2. Hardness

The hardness of the coatings was determined at five spots of the coating system surface, according to the EN ISO 868 standard [43]. The Shore durometer type D (LD0551, TQC Sheen, Molenbaan, The Netherlands)) was used to measure the hardness. The indenter of the durometer was pressed into the surface of the hardened coating material, and the hardness was determined using the durometer’s scale. The hardness of the coating system was determined for all topcoat layers of the coating systems—this layer will be exposed to mechanical stress in practice. Then, the results were compared with the reference coating materials.

### 3.3. Tensile Properties

The tensile properties (tensile strength and relative elongation at break) of each layer of the coating systems were determined according to the EN ISO 527-1 and EN ISO 527-2 standards [44,45]. Type 1B, double-sided, dog-bone-shaped test specimens hardened in silicone moulds were used for the determination of tensile properties.

### 3.4. Adhesion to the Substrate

This test was performed pursuant to the EN 4624 standard [46]. In order to perform this test, the coating systems were applied to concrete paving and metal substrates. The test output was the minimum tensile stress needed to rupture the weakest interphase (adhesive fracture) of the test layout. Metal targets with diameters of 20 mm were glued to the polymerised surfaces of the coating systems using a one-component cyanoacrylate adhesive. After the adhesive hardened in 24 h, the targets were cut with a cutting tool from the concrete or metal substrate. Then, the targets were pulled off using the Elcometer 506 pull-off adhesion tester.

### 3.5. Impact Resistance

This test was carried out pursuant to EN ISO 6272-1 [47]. The coating systems were applied on cement-bonded particle boards, then the cracking and peeling of the topcoat layers due to the deformation caused by a falling weight were monitored. The topcoat layer was tested, as it absorbs most of the mechanical stress, especially cyclic stress, in practice. Within the framework of this test, a large surface of a falling striker was chosen, since this method was suitable for the hard surfaces of the coatings and the higher elastic modulus of the coating materials.

### 3.6. Flexural Modulus

The flexural modulus of elasticity of each layer of the coating systems was determined according to the EN ISO 178 standard [48]. Three specimens from each layer were tested at a loading rate of 5 mm/min.

### 3.7. Abrasion Resistance

Specimens of the multilayer coating systems with dimensions of 71 × 71 mm were prepared for this test, and after seven days, the topcoat layer of each was subjected to the Böhme abrasion resistance test, according to the EN 13892-3 standard [49]. Abrasion resistance was determined as a reduction in volume calculated from the weight loss.

### 3.8. Determination of T_g_

The glass transition temperature (T_g_) was determined using the differential scanning calorimetry method (DSC) only on samples from the topcoat layers of the coating systems, which can be in contact with higher temperatures in practice. The softening temperature (T_m_) was determined on epoxy coating systems. The principle of the test was the heating of the ground specimen of the hardened material at a rate of 10 °C/min.

### 3.9. Chemical Resistance

The chemical resistance of the coating systems was determined using the accelerated aging test, which meant that the coating systems were exposed to chemical agents for 21 days. The chemical resistance test was performed on the coating systems that were applied on concrete pavement. Individual aggressive solutions acted on the surfaces of the coatings. They were allocated in polyethylene funnels, which were equipped with silicon, to avoid the leakage of liquid media. Specimens prepared in this manner were stored in a laboratory environment at a temperature of 23 °C for 21 days. The chemical agents chosen were aggressive substances (10% HCOOH, 30% HCl, 30% H_2_SO_4_) that the coatings may be exposed to in practice. Because the chemical resistance was determined by the accelerated aging test, the concentrations of the selected chemical agents were much higher than in practice.

The test was evaluated visually (disruptions, cracks, colour changes and coherence of layers) and by using the Keyence VHX950F digital optical microscope (Keyence Ltd., Osaka, Japan) with magn. up to 63.7×. Next, the chemical resistance was compared with the reference coating systems. After chemical stress, hardness and adhesion to the substrate were examined on the loading coating systems, and the results of these tests were compared with the values acquired prior to the chemical stress. Furthermore, the microstructure of the materials exposed to the chemically aggressive environment was monitored using the TESCAN MIRA3 XMU scanning electron microscope (SEM) at up to 10,000× resolution. The distribution of the microfillers in the matrix and the effect of the aggressive solutions on the resistance of the filler itself were monitored as well.

## 4. Results and Discussion

### 4.1. Dynamic Viscosity

**Figure 6:**
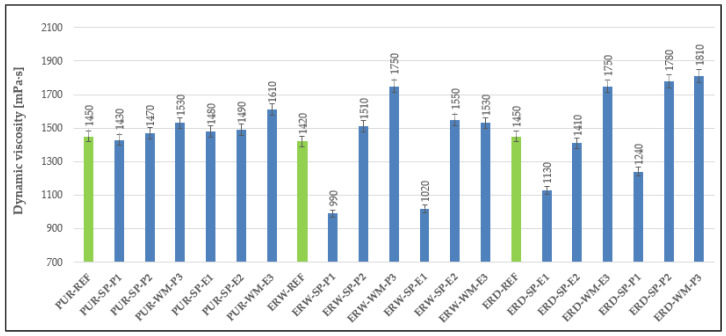
Dynamic viscosity of materials forming the individual layers of the coating systems.

The results of the dynamic viscosity determination are stated in Figure 6. For epoxy-based coatings, it is noticeable that dynamic viscosity increased with higher amounts of fillers. There was no significant difference in the dynamic viscosity of the materials that made up the individual layers of the polyurethane coating systems, which is related to the fact that the amount of filler in each layer was the same. The composition of the individual microfillers had no significant effect on the coating materials’ dynamic viscosity. It can be said that the dynamic viscosity was influenced mainly by the type of resin used. The highest viscosities were recorded in the formulations in which the topcoat layers of the coating systems were based on epoxy resins (ERD, ERW).

### 4.2. Hardness

**Figure 7:**
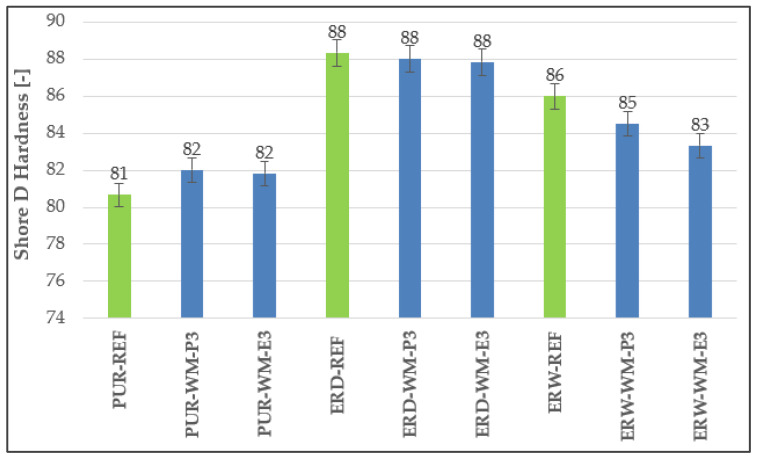
Surface hardness of the polymer coating systems.

In accordance with the assumptions, the highest surface hardness can be observed for the topcoats of the ERW coating systems. Figure 7 shows that the hardness of the CS-P coating system was slightly higher than CS-E. The hardness of the polyurethane-based coating systems, which contained secondary raw materials in the topcoat layers, was even higher than the reference materials. Generally, epoxy-based coating materials have better mechanical properties than polyurethane materials; however, the low hardness values of the PUR materials may be caused by the lower filler content, namely 20 wt%. In the epoxy-based materials, a filler in the amount of 30 wt% was used. Hence, it can be seen that the surface hardness of the coating increased with the higher content of filler used in the polymer matrix. Memon et al. [50] found out that the inclusion of fillers resulted in up to 38 points increase in Shore D hardness relative to the pristine polyurethane coatings.

### 4.3. Tensile Properties

**Figure 8:**
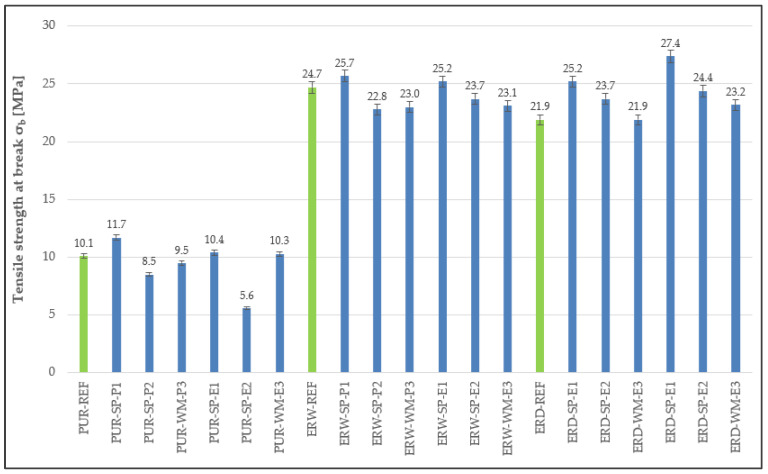
Tensile strength at break of the individual layers forming the coating system.

**Figure 9:**
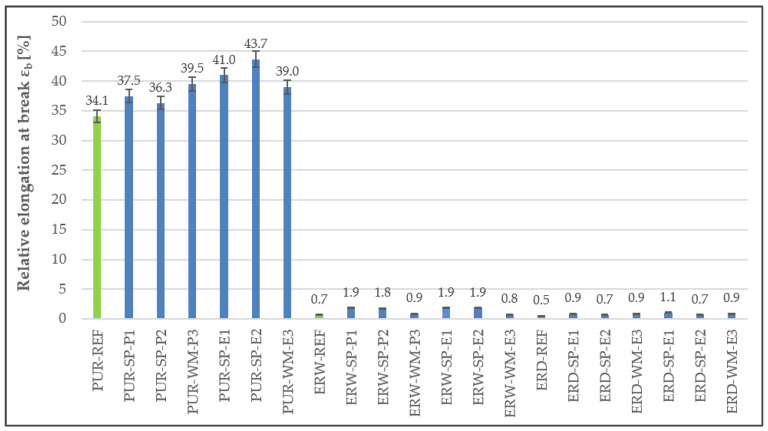
Relative elongation at break of the individual layers forming the coating system.

Upon the determination of the tensile properties of the individual layers of the coating systems, the highest tensile strength was observed in materials based on solvent-free epoxy resin (see Figure 8). Specifically, the highest value, which even significantly exceeded the reference material values, was observed in the first layer with the SP-E1 filler. It is clear from the tensile properties that dependency on the type and amount of filler is not significant, and thus tensile properties depend mainly on the type of polymer binder used. The lowest tensile strength was observed in the polyurethane coating systems. However, if there is an opening crack in a concrete substrate, high relative elongation is desirable to bridge the crack without breaking. Thus, dependency between tensile strength and relative elongation (Figure 9) can be observed, which means that the higher the tensile strength, the lower the elongation upon breaking. Tensile properties, the same as high shear strength, in un-cross-linked polymers are directly related to high zero-shear-rate viscosity. Shear resistance can be substantially raised when polyurethane is incorporated into the acrylic network, indicating an increase in film cohesion [29].

According to Manigandan’s results, the addition of polyurethane-coated specimens along with fibres showed better flexural strength and decreased stiffness. The addition of 11% and 5.2% filler led to a reduction in compressive and tensile strength [22]. Krzywiński et al. [51] discovered that the addition of recycled fine aggregate (containing 88% SiO_2_) to an epoxy resin coating did not affect the flexural, tensile and compressive strength of the polymer composite.

### 4.4. Adhesion to the Substrate

The adhesion of the coating systems to the metal surface was significantly lower than to the concrete surface. The low adhesion to metal was also influenced by the fact that the metal substrate was galvanised sheet metal. It can be assumed that the coating systems would have higher adhesion to a metal substrate without treatment.

**Figure 10:**
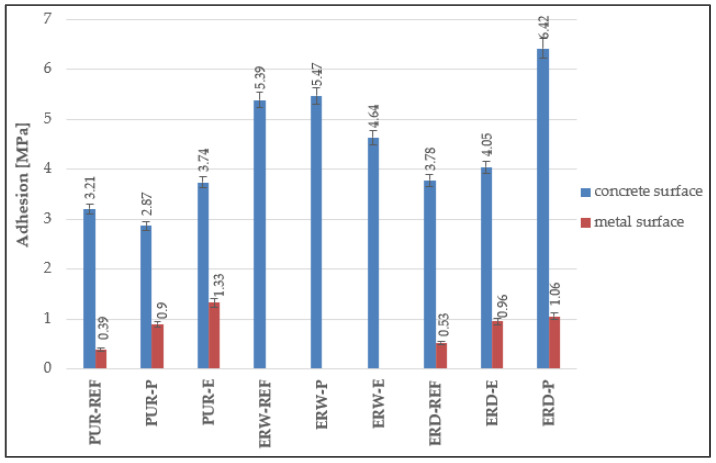
Adhesion of the coating systems to the concrete and metal substrate.

It can be seen from Figure 10 that the highest adhesion to the concrete surface was observed for the CS-E coating system. In all tested coating systems, the adhesion to concrete was higher than 2.0 MPa, and the disruption occurred in the substrate or in the adhesive. The ERD-P coating system showed even higher adhesion—higher than 6.0 MPa—from which it can be concluded that the coherence of the coatings with concrete substrates is exceptional and that coating systems with incorporated HW will be able to protect concrete construction even in the long term. According to Chowaniec et al. [30], replacing a part of the epoxy resin by silica flour the range of 5–40 μm can reduce the amount of hazardous substances in the coating (e.g., bisphenol A) and the cost, and it is possible to retain or improve coating adhesion on concrete surfaces. Adhesion strength refers to the mutual adhesion between different surfaces which relies on the interfacial interactions [52].

### 4.5. Impact Resistance

**Figure 11:**
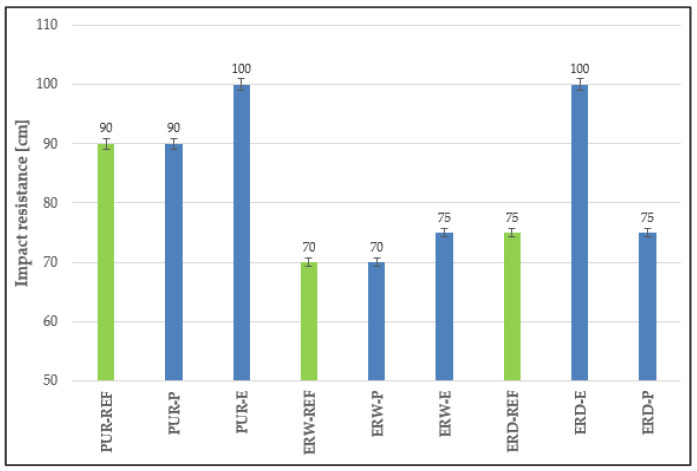
Impact resistance of the coating systems.

It is obvious from Figure 11 that the highest impact resistance was found for the polyurethane resin-based coating systems. PURs are usually more flexible, and their elastic modulus is lower, and so they are less brittle (more impact resistant) than epoxy coating systems, i.e., they can better absorb potential energy.

### 4.6. Flexural Modulus

**Figure 12:**
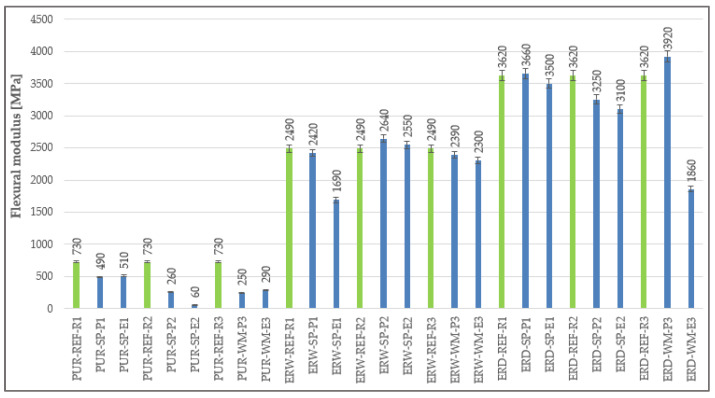
Flexural modulus of elasticity of the individual layers of coating systems.

The highest flexural modulus of elasticity was observed in the materials based on solvent-free epoxy resin (ERD). Compared with the other materials, the CS-E coating system showed lower flexural modulus in the last layer, in which only fly ash was used as a filler (see Figure 12). The lowest flexural modulus was observed in PUR-based coating systems, as polyurethane resins generally belong to the family of the most flexible resins. Closely related to these results were the observed values of impact resistance and tensile properties of the coating materials. Due to the fact that the PUR coating materials had the lowest flexural modulus, they showed higher impact resistance, thanks to lower brittleness.

### 4.7. Abrasion Resistance

**Figure 13:**
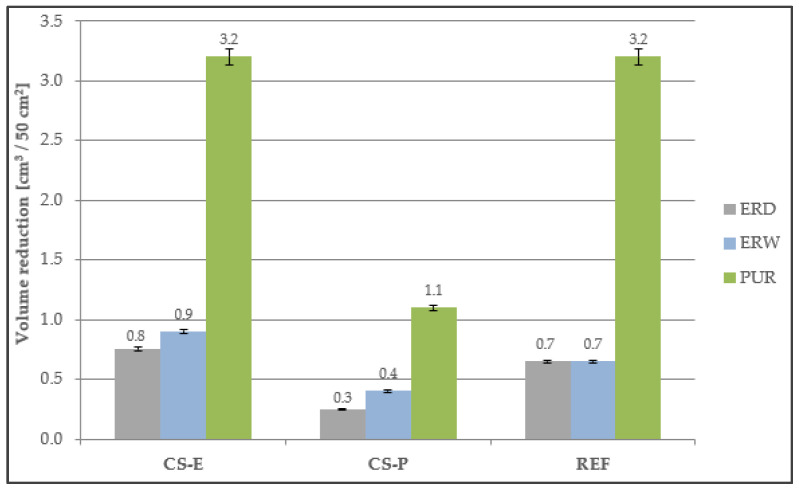
Abrasion resistance of the coating systems.

Based on the results of the abrasion resistance test (Figure 13), it can be stated that the PUR-based coating systems showed the lowest abrasion resistance. This is related to the elastic modulus, which was very low for PUR. Of the epoxy resins, the coatings based on solvent-free ERD were more resistant to abrasion. It can be seen that all coating systems in the CS-P quality class had lower volume reductions than the reference coating materials. Therefore, abrasion resistance was influenced not only by the binders used but also by the fillers used. The resistance of top coats against abrasive wear is an important lifetime parameter for corrosion protection coating systems [53]. The main characteristics of abrasive wear is about plowing or cutting of the coating’s surface by a harder material [54]. Barbakadze et al. [55] stated that the need for new epoxy coatings with improved abrasion resistance and lower friction coefficients is a very active field nowadays. Regarding this statement, the new developed coating systems with high abrasion resistance, containing also HW, will also be in high demand in practice.

### 4.8. Determination of T_g_

**Figure 14:**
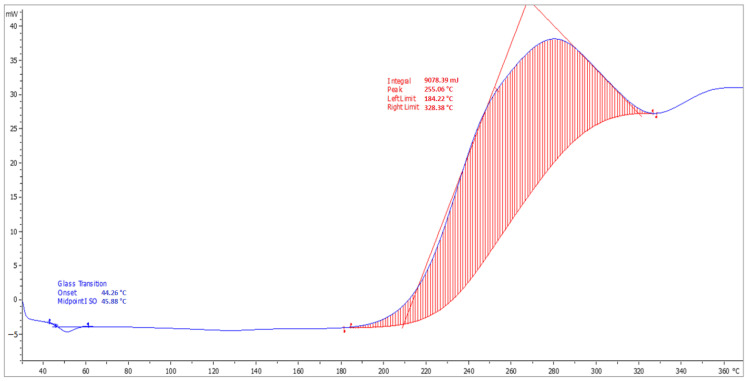
Record from DSC analysis with indications of T_g_ and T_m_ temperatures of the coating layer ERD-P3.

**Figure 15:**
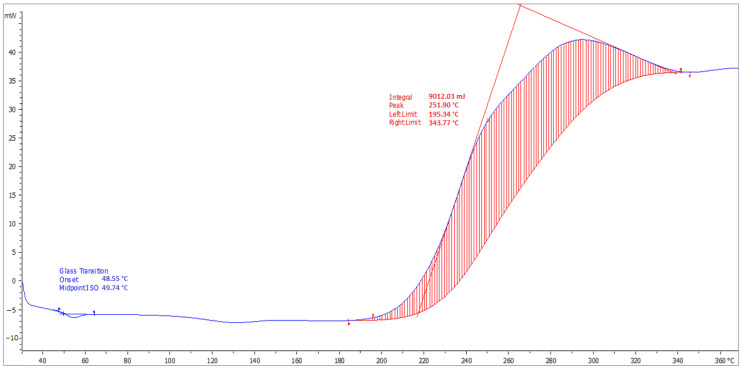
Record from DSC analysis with indications of T_g_ and T_m_ temperatures of the coating layer ERW-P3.

**Figure 16:**
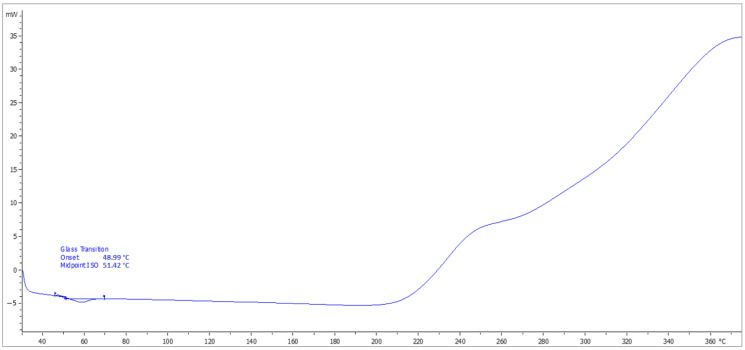
Record from DSC analysis with indication of T_g_ of the coating layer PUR-P3.

**Figure 17:**
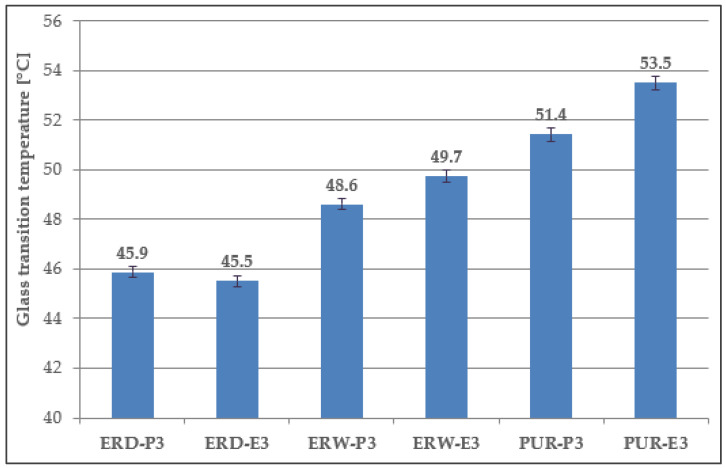
Dependence of T_g_ on the type of coating.

The DSC analysis records with indications of the glass transition temperature (T_g_) and softening temperature (T_m_) of the top coats of the coating materials (ERD-P, ERW-P, PUR-P) are stated in Figure 14, Figure 15 and Figure 16. The graphical comparison of the T_g_ values for the topcoat layers is stated in Figure 17. The highest T_g_ values were observed in the materials based on polyurethane resin. Lower T_g_ values were observed in the coatings based on solvent-free epoxy resin. It follows from the results that the filler type has only a negligible influence on the T_g_ value.

Yeasmin et al. [56] concluded in their research that it is possible to obtain a higher glass transition temperature of epoxy resin with the addition of a silica. From the results of the determination of T_g_, it is clear that the difference between the CS-P and CS-E coating systems using the same polymer binders was not significant.

### 4.9. Chemical Resistance

#### 4.9.1. Evaluation by Digital Optical Microscopy

No colour change, creation of blisters or peeling of the coating system off the substrate occurred in the ERD-REF material exposed to a solvent (xylene) and 30% H_2_SO_4_. When exposed to 30% HCl, only a colour change occurred, but when exposed to 10% HCOOH, a significant colour change and creation of blisters occurred.

**Figure 18:**
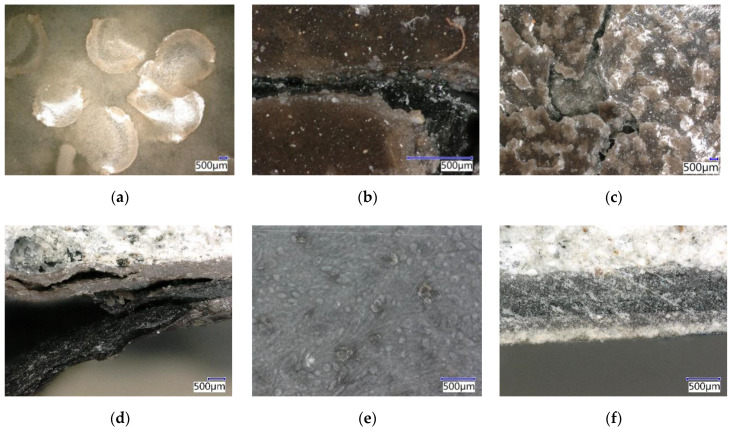
Epoxy coating systems after exposure to the chemically aggressive environment: (**a**) Surface of ERD-REF after exposure 10% HCOOH (magn. 6.4×); (**b**) Cracked ERD-E after exposure 10% HCOOH (magn. 63.7×); (**c**) Surface ERD-P after exposure 10% HCOOH (magn. 6.4×); (**d**) ERD-E after exposure to 10% HCOOH (magn. 16×); (**e**) Surface of ERD-P after exposure 30% H_2_SO_4_ (magn. 31.8×); (**f**) ERD-P after exposure to 30% HCl (magn. 31.8×).

As well as the reference coating system (Figure 18a), the ERD-E (Figure 18b) and ERD-P (Figure 18c) coating systems showed degradation upon exposure to 10% HCOOH. There were disruptions, namely cracks in the topcoat (sealing) layers, and the aggressive acid solution came in contact with the lower layers of the coating systems through the cracks. It is possible to assume that after longer exposure to 10% HCOOH, the degradation of other layers of the coating systems would occur as well. As seen in Figure 18d, which illustrates the cut of the ERD-E system, this degradation effect was already present at some spots, with obvious disruptions in all three layers. The same degradation occurred in the ERD-P coating system. The exposure to 30% HCl only caused the lightening and smoothing of the surface of the coating system. As with ERD-REF, the surface of ERD-P and ERD-E showed no significant changes after exposure to 30% H_2_SO_4_ (Figure 18e). The ERD-E specimen was slightly less resistant to the solvent than ERD-REF. After exposure to 30% HCl, there was only a visual change, namely a lightening of the surface. The cut through the intact three-layer coating system can be seen in Figure 18f.

**Figure 19:**
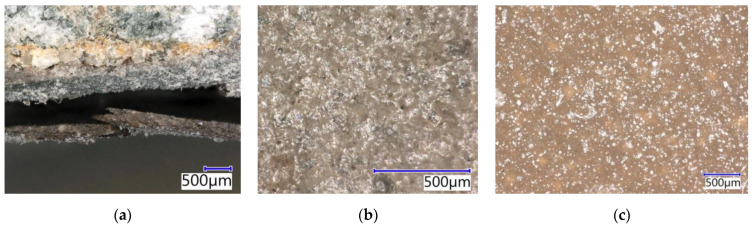
Epoxy coating systems after exposure to the chemically aggressive environment: (**a**) ERW-E after exposure to 10% HCOOH (magn. 16×); (**b**) ERW-E surface after exposure to xylene (magn. 63.7×); (**c**) ERW-P surface after exposure to 30% H_2_SO_4_ (magn. 31.8×).

Based on the images from the digital optical microscope, it is possible to assume that the chemical resistance of ERW-E (Figure 19a) was similar to the solvent-free ERD coating systems. After exposure to 10% HCOOH and xylene, a degradation of the coating system and the creation of blisters occurred (Figure 19b). Upon exposure to H_2_SO_4_ and HCl, only a colour change of the coating occurred (Figure 19c).

Based on the findings above, it can be assumed that the tested epoxy-based coating systems will be resistant to inorganic acids, such as concentrated H_2_SO_4_ and HCl. Their high resistance is expected within the framework of their intended application, e.g., the sewage system.

**Figure 20:**
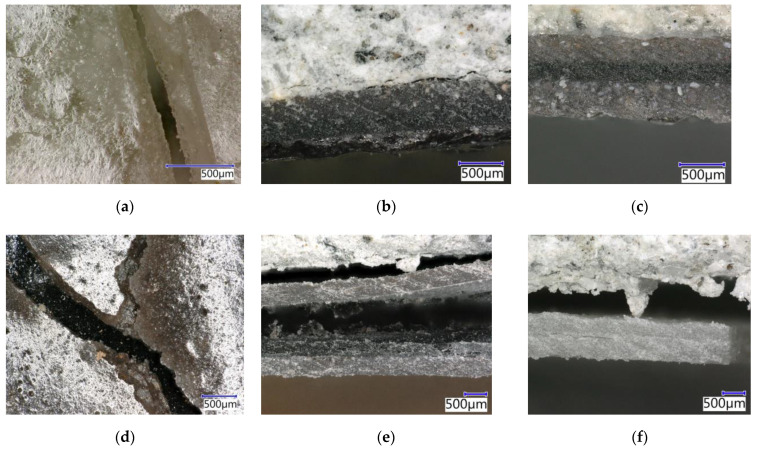
Polyurethane coating systems after exposure to the chemically aggressive environment: (**a**) Crack in PUR-REF after exposure xylene (magn. 63.7×); (**b**) PUR-E after exposure to 10% HCOOH (magn. 31.8×); (**c**) Cut of PUR-E after exposure 30% H_2_SO_4_ (magn. 31.8×); (**d**) Damaged topcoat layer of PUR-E after exposure to xylene (magn. 31.8×); (**e**) PUR-E after exposure to xylene (magn. 16×); (**f**) PUR-REF after exposure to xylene (magn. 16×).

Only a colour change occurred in the polyurethane coating systems after exposure to 30% HCl. A crack in the coating system with a width of about 500 µm, caused by the action of xylene, is visible in Figure 20a. The resistance to 10% HCOOH was better than in epoxy-based coating systems, but a loss of cohesion with the substrate was also observed (Figure 20b) even though no cracks were visible in the individual layers. The aggressive HCOOH solution probably penetrated through the individual cracks to the concrete, which resulted in a bland separation that was invisible to visual evaluation. Due to the exposure to 10% HCOOH, the polyurethane coating system also softened. No significant damage to the coating surface was caused by sulphuric acid, only a weak penetration of the acid into the surface structure (Figure 20c) The exposure to the solvent resulted in complete degradation and the disruption of the adhesion of the coating to the substrate and the individual layers (Figure 20d–f).

Regarding the evaluation of chemical resistance, it can be claimed that the influence of the fillers in the form of solidification products had no negative impact on the chemical resistance of the polymer coating systems, nor was there any release of the fillers from the polymer matrix. It can be assumed that the pollutants remained incorporated into the polymer matrix even after exposure to a strongly aggressive chemical environment. The coating systems with incorporated HW were almost as resistant to the chemical agents as the reference coating systems. When used in practice, it can be mainly expected that the developed epoxy-based coating systems will not resist weak organic acids but will resist strong inorganic acids and solvents. By contrast, the PUR-based coating systems will be more resistant to weak organic acids but less to solvents in practice. As expected, chemical resistance depended on the type of polymer binder used. There was also a difference between the chemical resistance of the coating systems, CS-P and CS-E. The chemical resistance of the coating system was affected not only by the binder but also the type of filler to a lesser extent. The objective was to reach the highest possible chemical resistance of the CS-P coating systems, which was proven in the chemical resistance test.

Non-toxic and renewable materials such as the agro-industrial waste were studied as alternative that may reduce or replace the use of epoxy resin and that can provide coatings with hydrophobicity and flexibility, improving the anticorrosive properties. It was proven by de Silva et al. [57] that bio-based epoxy coatings with the content of agro-industrial waste presented a very good balance between flexibility and wettability properties along with excellent chemical resistance, thermal stability and anti-corrosion performance.

#### 4.9.2. Hardness after Chemical Stress

**Figure 21:**
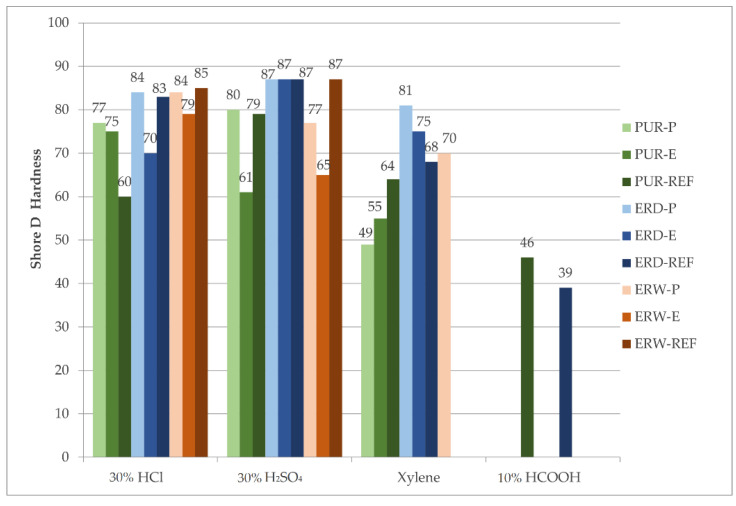
Hardness of the coating systems after chemical stress.

Due to the total degradation of the coating system, it was impossible to determine the hardness for some specimens, e.g., those exposed to 10% HCOOH and xylene. The results of the Shore D hardness test of the coating systems exposed to chemically aggressive environments are stated in Figure 21.

The smallest average decrease in hardness occurred for the CS-P coating system. In Figure 21, the greatest influence on the degradation of the coating systems, and thus on the reduction in hardness, are shown for 10% HCOOH. By contrast, the least influence on the reduction in the hardness of the coating systems was for 30% H_2_SO_4_. For solvent-free ERD coating systems, there was almost no decrease in hardness when exposed to sulphuric acid.

#### 4.9.3. Adhesion to Concrete after Chemical Stress

For most specimens, the failure after the test occurred in the adhesive because the surface of the coating system became smoother, and the solution crystallised due to the influence of chemically aggressive solutions, mainly in form of acids. Hence, the adhesion of the adhesive used to glue the targets on the coating’s surface decreased. However, in most cases, when the coating was not seriously damaged, it was possible to perform the tests and determine the change in the adhesion to the concrete substrate. The results, which include the adhesion change compared with unstressed samples and the spots of disruption, are stated in Table 6.

The highest adhesion was obtained for the CS-P epoxy-based coating systems. These values were significantly higher than for the reference coating materials. The ERW-based coating systems showed slightly lower values, and most of the samples could not be tested. Significantly better adhesion was observed for the PUR-based coating systems. The results of the adhesion test were closely related to the results of the hardness and microscopical evaluations after chemical stress. Interestingly, although the polyurethane coating system’s exposure to 10% HCOOH resulted in a slight separation of the coating system from the substrate at some spots (Figure 20b), the adhesion to concrete was nevertheless higher than 2.0 MPa. Based on the results, it can be assumed that exposure to strong inorganic acids will not reduce the adhesion of the coating system to concrete in practice.

#### 4.9.4. Microstructure after Chemical Stress by Using SEM

**Figure 22:**
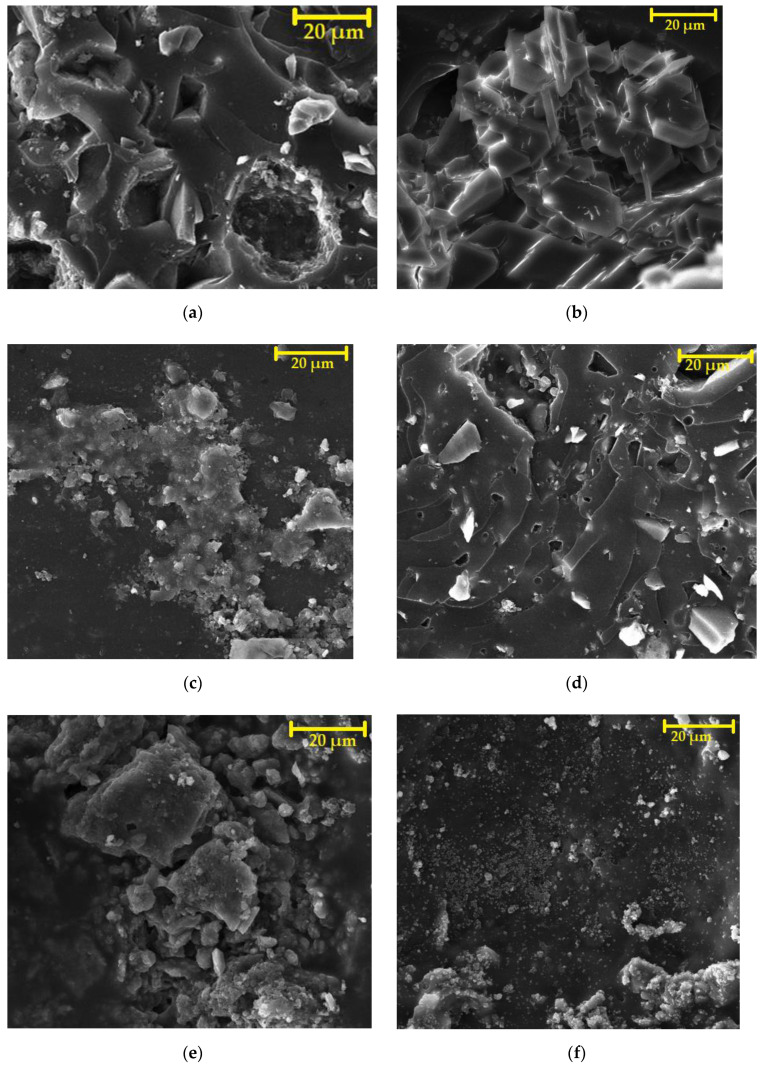
SEM photomicrographs of the coating systems (magn. 2000×): (**a**) primer of ERD-P stressed by 10% HCOOH connected to concrete; (**b**) degraded topcoat layer of ERD-P stressed by 10% HCOOH; (**c**) degraded topcoat layer of ERD-REF stressed by 10% HCOOH; (**d**) primer of ERW-P stressed by 10% HCOOH; (**e**) degraded topcoat layer of PUR-E sample stressed by 10% HCOOH; (**f**) primer of PUR-P, connected to concrete, stressed by xylene.

The microstructure of the epoxy-based ERD-P coating system after exposure to 10% HCOOH can be seen in Figure 22a,b. In Figure 22a, where the primer connected to concrete is shown, the disturbance of integrity can be seen although this layer was not in contact with the aggressive medium at first. Newly formed open pores can be seen here due to the penetration of 10% HCOOH even to this layer, which was the closest one to the substrate. Figure 22b shows crystals formed by the exposure to 10% HCOOH in the topcoat layer, which was in direct contact with this aggressive acid. It is likely that the filler (solidification product) located in the polymer matrix reacted with the HCOOH. CaO occurred in the fillers due to the content of both hazardous waste (cement bypass dust, end product) and used fly ash. As a result of the reaction with formic acid, CaO may have reacted to form calcium formate (1).

2HCOOH + CaO → (HCOO)_2_Ca + H_2_O (1)

Carboxylic acids react with epoxy groups to form β-hydroxy propyl ester, which, in turn, reacts with another carboxylic acid to yield a diester. The hydroxyl ester can also undergo polymerisation by the reaction of the secondary hydroxyl group with the epoxy [58].

The microstructure of the reference coating system specimen, which was also chemically stressed by 10% HCOOH for 28 days, is illustrated in Figure 22c. However, in this case, no crystals were formed by the action of carboxylic acids. The degradation of the individual layers consisted of a violation of the structures of the coating layers of the protection system, known as ‘delamination’. In Figure 22d, the degradation can be observed, i.e., the disturbance of the first layer of the ERW-P coating system, which shows the principle of loss of functionality of the coating system by the disintegration and subsequent punctures of the resulting microlayers of the coating. Upon longer exposure to 10% HCOOH, a total degradation would occur even in the first layer of the coating system based on the waterborne epoxy resin.

Using the SEM, no influence on the inner structure and microstructure of the tested coating system exposed to strong solutions of inorganic acids (HCl, H_2_SO_4_) was proven—their microstructure was identical to specimens not stressed by a chemically aggressive environment.

Figure 22e,f show the microstructure of the polyurethane coating systems after applying 10% HCOOH solvent (xylene) on their surfaces. The exposure of the microfiller particles or crystals present in the filler can be seen in the polyurethane fillers. In this case, the degradation was caused by two mechanisms, namely the cracking of the coatings’ layers and the washing off of the PUR resin around the microfiller particles that disrupted the contact zone between the filler and the binder. Consequently, the coating system degraded and ceased to fulfil its protective function after exposure to the solvent.

**Figure 23:**
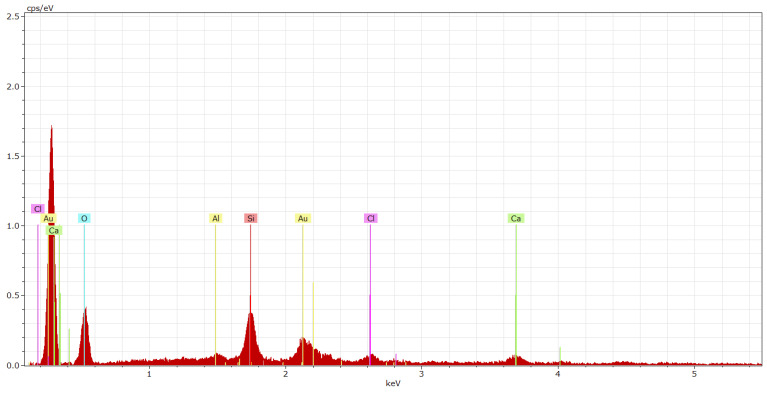
EDX spectrum of the ERD-P coating system (topcoat layer) stressed by 10% HCOOH.

**Figure 24:**
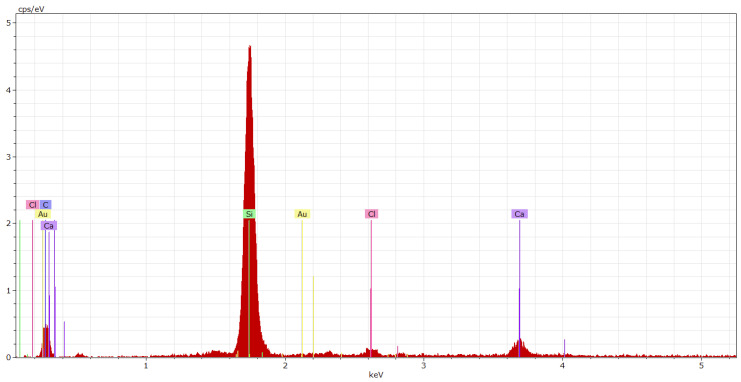
EDX spectrum of the PUR-P coating system (primer layer connected to concrete) stressed by xylene.

EDX of the coating systems was carried out at spots where the crystal phases were noticeable when observed using the SEM. Thanks to the EDX analysis and the SEM photomicrographs, it was possible to state that the filler containing pre-treated HW was evenly distributed in the polymer matrix. In Figure 23 and Figure 24, elements such as carbon and oxygen represent the resin itself, which encapsulates the individual particles of the microfillers that are represented by elements such as Ca, Si and Al. Chlorines represent particles of HW (cement bypass dust) solidified by the polymer matrix of the coating system.

## 5. Conclusions

The physical and mechanical properties and the chemical resistance, supported by the microstructure analysis, of newly developed coating systems containing specific HW (end product and cement bypass) were verified. Pre-treated HW was successfully incorporated into epoxy and polyurethane coating systems. It was proven that coating systems that contain HW show the same and, in some cases, even better physical and mechanical properties (e.g., adhesion, hardness, tensile properties, etc.) than the reference coating systems that use only primary raw materials. The progressive filler with HW did not negatively influence the coherence of individual layers or adhesion to the substrate. The type of HW used also had no significant effect on the mechanical properties of the coating systems. In terms of chemical resistance, the developed coating systems with HW withstood the aggressive chemical environment and tested almost equally to the reference coating systems. The degradation of the coating systems after exposure to 10% HCOOH, with regard to the change in their microstructure, was closely monitored, while the degradation mechanism of the coating systems tested was clarified. Using the SEM, it was observed that HW particles were firmly incorporated into the polymer matrix. In places where the coating systems containing HW are to be used (e.g., sewage systems), protection will be ensured, as the pollutants from the HW will not leak into the liquid environment even in a strongly aggressive environment.

## Figures and Tables

**Table 1:** Composition of solidification products used as microfillers in wt%.

Coating System	Filler	Cement Bypass Dust ^1^	End Product ^1^	FC	FF	Silica Flour
CS-E	SP-E1	10	-	90	-	-
	SP-E2	5	-	-	95	-
	WM-E3	-	-	100	-	-
CS-P	SP-P1	-	10	40	-	50
	SP-P2	-	5	-	35	60
	SP-E1	-	-	40	-	60

^1^ Hazardous waste.

**Table 2:** Chemical composition of the polymer binders.

Binder	Component A	Component B
Solvent-free epoxy resin (ERD)	Epoxy resin with average molecular weight ≤ 700	Formaldehyde
	(Alkoxymethyl) oxirane (alkyl C12-C14) formaldehyde	Polymer with N’-(3-aminopropyl)propane-1,3-diamine
	Oligomeric reaction products with 1-chloro-2,3-epoxypropane and phenol	polyamine adduct, carbomonocyclic alkylated mixtures of poly-aza-alkanes, hydrogenated
Water-soluble epoxy resin (ERW)	Epoxy resin with average molecular weight ≤ 700	fatty acids, tall oil, reaction product with tetraethylenetriamine
	(Alkoxymethyl) oxiran (alkyl C12-C14) formaldehyde	4,4-Isopropylidenediphenol, 3-Aminopropyldimethylamine
	Oligomeric reaction products with 1-chloro-2,3-epoxypropane and phenol	2,4,6-Tris(dimethylaminomethyl)phenol, 1,3-bis(aminomethyl)benzene
Polyurethane resin (PUR)	Fumaric acid diethyl ester	Hexamethylene diisocyanate
		oligomers

**Table 3:** Formulations with the epoxy binder ERD.

Type of Coating System	Layer	Polymer Binder	Filler	Filler Content [wt%]
CS-E	1	ERD	SP-E1	20
	2	ERD	SP-E2	30
	3	ERD	WM-E3	30
CS-P	1	ERD	SP-P1	20
	2	ERD	SP-P2	30
	3	ERD	WM-P3	30

**Table 4:** Formulations with the epoxy binder ERW.

Type of Coating System	Layer	Polymer Binder	Filler	Filler Content [wt%]
CS-E	1	ERW	SP-E1	30
	2	ERW	SP-E2	30
	3	ERW	WM-E3	30
CS-P	1	ERW	SP-P1	30
	2	ERW	SP-P2	30
	3	ERW	WM-P3	30

**Table 5:** Formulations with polyurethane binder (PUR).

Type of Coating System	Layer	Polymer Binder	Filler	Filler Content [wt%]
CS-E	1	PUR	SP-E1	20
	2	PUR	SP-E2	20
	3	PUR	WM-E3	20
CS-P	1	PUR	SP-P1	20
	2	PUR	SP-P2	20
	3	PUR	WM-P3	20

**Table 6:** Adhesion of the coating systems to concrete after chemical stress.

Coating System	Solution	Adhesion [MPa]	Reduction in Adhesion [%]	Place of Failure
ERD-P	30% HCl	3.71	42.2	glue
	30% H_2_SO_4_	3.57	44.4	glue
	xylene	4.01	37.5	concrete
ERD-E	xylene	4.01	37.5	concrete
	30% HCl	4.15	1.0	glue
	30% H_2_SO_4_	4.00	1.23	glue
	xylene	2.37	41.5	glue
ERD-REF	30% HCl	1.04	72.5	glue
	10% HCOOH	0.57	86.8	glue
ERW-P	30% HCl	3.16	42.3	glue
ERW-E	30% HCl	3.01	35.1	glue
PUR-P	30% HCl	3.68	1.0	concrete
	30% H_2_SO_4_	2.07	27.9	glue
	10% HCOOH	2.68	6.6	concrete
PUR-E	30% HCl	1.78	52.4	glue
	10% HCOOH	3.48	6.6	concrete
PUR-REF	30% HCl	0.12	96.3	concrete
	30% H_2_SO_4_	3.07	4.4	concrete
	10% HCOOH	3.01	6.2	glue

## Data Availability

Not applicable.

## References

[1] Zhang R., Mallon P.E., Chen H., Huang C.M., Zhang J., Li Y., Jean Y.C. (2001). Characterization of photodegradation of a polyurethane coating by positron annihilation spectroscopy: Correlation with cross-link density. Prog. Org. Coat..

[2] Clough R.L., Billingham N.C., Gillen K.T. (1996). Polymer Durability: Degradation, Stabilization, and Lifetime Prediction, Ser. No. 249.

[3] Sadowski Ł., Kampa Ł., Chowaniec A., Królicka A., Żak A., Abdoulpour H., Vantadori S. (2021). Enhanced adhesive performance of epoxy resin coating by a novel bonding agent. Constr. Build. Mater..

[4] Almusallam A.A., Khan F.M., Dulaijan S.U., Al-Amoudi O.S.B. (2003). Effectiveness of surface coatings in improving concrete durability. Cem. Concr. Compos..

[5] Makki H., Adema K.N.S., Peters E.A.J.F., Laven J., van der Ven L.G.J., van Benthem R.A.T.M., de With G. (2014). A simulation approach to study photo-degradation processes of polymeric coatings. Polym. Degrd. Stab..

[6] Ahmetli G., Deveci H., Soydal U., Seker A., Kurbanli R. (2012). Coating, mechanical and thermal properties of epoxy toluene oligomer modified epoxy resin/sepiolite composites. Prog. Org. Coat..

[7] Chowaniec A., Sadowski Ł., Żak A. (2020). The chemical and microstructural analysis of the adhesive properties of epoxy resin coatings modified using waste glass powder. Appl. Surf. Sci..

[8] Atta A.M., Abdou M.I., Elsayed A.A., Ragab M.E. (2008). New bisphenol novolac epoxy resins for marine primer steel coating applications. Prog. Org. Coat..

[9] Liu M., Mao X., Zhu H., Lin A., Wang D. (2013). Water and corrosion resistance of epoxy-acrylic-amine waterborne coatings: Effects of resin molecular weight, polar group and hydrophobic segment. Corros. Sci..

[10] Li L., Yu Y., Wu Q., Zhan G., Li S. (2009). Effect of chemical structure on the water sorption of amine-cured epoxy resins. Corros. Sci..

[11] Chen P., Wang Y., Li J., Wang H., Zhang L. (2018). Adhesion and erosion properties of epoxy resin composite coatings reinforced with fly ash cenospheres and short glass fibers. Prog. Org. Coat..

[12] Dulaijan S.U., Maslehuddin M., Al-Zahrani M.M., Al-Juraifani E.A., Alidi S.A., Al-Meththel M. Performance evaluation of cement- based surface coatings. Proceedings of the 2000 International Conference, Repair, Rehabilitation and Maintenance of Concrete Structures and Innovations in Design and Construction.

[13] Santos J.C., Vieira L.M.G., Panzera T.H., Schiavon M.A., Christoforo A.L., Scarpa F. (2015). Hybrid glass fibre reinforced composites with micro and poly-diallyldimethylammonium chloride (PDDA) functionalized nano silica inclusions. Mater. Des..

[14] Kasaeian M., Ghasemi E., Ramezanzadeh B., Mahdavian M., Bahlakeh G. (2018). Construction of a highly effective self-repair corrosion-resistant epoxy composite through impregnation of 1H-Benzimidazole corrosion inhibitor modified graphene oxide nanosheets (GO-BIM). Corros. Sci..

[15] Heydarpour M.R., Zarrabi A., Attar M.M., Ramezanzadeh B. (2014). Studying the corrosion protection properties of an epoxy coating containing different mixtures of strontium aluminum polyphosphate (SAPP) and zinc aluminum phosphate (ZPA) pigments. Prog. Org. Coat..

[16] Ruiz M.M., Cavaillé J.Y., Dufresne A., Graillat C., Gérard J. (2001). New waterborne epoxy coatings based on cellulose nanofillers. Macromol. Symp..

[17] Noreen A., Zia K.M., Zuber M., Tabasum S., Saif M.J. (2016). Recent trends in environmentally friendly water-borne polyurethane coatings: A review. Korean. J. Chem. Eng..

[18] Wegmann A. (1997). Chemical resistance of waterborne epoxy/amine coatings. Prog. Org. Coat..

[19] Atta A.M., Shaker N.O., Nasser N.E. (2007). Synthesis of bisphenol a novolac epoxy resins for coating applications. J. Appl. Polym. Sci..

[20] Li J., Cui J., Yang J., Li Y., Qiu H., Yang J. (2016). Reinforcement of graphene and its derivatives on the anticorrosive properties of waterborne polyurethane coatings. Compos. Sci. Technol..

[21] Andersson C. (2008). New ways to enhance the functionality of paperboard by surface treatment–a review. Packag. Technol. Sci..

[22] Manigandan S., Praveenkumar T.R., Al-Mohaimeed A.M., Brindhadevi K., Pugazhendhi A. (2021). Characterization of polyurethane coating on high performance concrete reinforced with chemically treated Ananas erectifolius fiber. Prog. Org. Coat..

[23] Boisaubert P., Kébir N., Schuller A.S., Burel F. (2020). Photo-crosslinked coatings from an acrylate terminated non-isocyanate polyurethane (NIPU) and reactive diluent. Eur. Polym. J..

[24] Tao Y., Sun G., Wei Y., Liu R., Zhao J. (2021). An anti-shrinkage model of an ultraviolet-curing coating filled with hollow polyurethane acrylate microspheres. Mech. Mater..

[25] Mohanty S.R., Mohanty S., Nayak S.K. (2021). Synthesis and evaluation of novel acrylic and ester-based polyols for transparent polyurethane coating applications. Mater. Today Commun..

[26] Wang H., Xu J., Du X., Du Z., Cheng X., Wang H. (2021). A self-healing polyurethane-based composite coating with high strength and anti-corrosion properties for metal protection. Compos. B Eng..

[27] Yang H., Zhang M., Chen R., Liu Q., Liu J., Yu J., Zhang H., Liu P., Lin C., Wang J. (2021). Polyurethane coating with heterogeneity structure induced by microphase separation: A new combination of antifouling and cavitation erosion resistance. Prog. Org. Coat..

[28] Qian Y., Dong F., Guo L., Xu X., Liu H. (2021). Two-component waterborne polyurethane modified with terpene derivative-based polysiloxane for coatings via a thiol-ene click reaction. Ind. Crops. Prod..

[29] Lopez A., Degrandi-Contraires E., Canetta E., Creton C., Keddie J.L., Asua J.M. (2011). Waterborne polyurethane-acrylic hybrid nanoparticles by miniemulsion polymerization: Applications in pressure-sensitive adhesives. Langmuir.

[30] Chowaniec A., Czarnecki S., Sadowski Ł. (2021). The effect of the amount and particle size of the waste quartz powder on the adhesive properties of epoxy resin coatings. Int. J. Adhes. Adhes..

[31] Yogeshwaran S., Natrayan L., Udhayakumar G., Godwin G., Yuvaraj L. (2021). Effect of waste tyre particles reinforcement on mechanical properties of jute and abaca fiber-epoxy hybrid composites with pre-treatment. Mater. Today Proc..

[32] Sharma V., Meena M.L., Kumar M., Patnaik A. (2021). Optimization of waste fly ash powder filled glass fiber reinforced epoxy composite by hybrid AHP-TOPSIS approach. Mater. Today Proc..

[33] Borsaikia A.C., Kumar A., Raj A., Dixit U.S. (2020). Development of epoxy based composites using bamboo and waste metal chips. Encycl. Renew. Sustain. Mater..

[34] Dalhat M.A. (2020). Utilization of date pits waste as aggregate alternative in sand-epoxy-resin composite. Constr. Build. Mater..

[35] Sevinç A.H., Durgun M.Y. (2021). A novel epoxy-based composite with eggshell, PVC sawdust, wood sawdust and vermiculite: An investigation on radiation absorption and various engineering properties. Constr. Build. Mater..

[36] Hegde S., Padmaraj N.H., Siddesh V., Sunaya T.S., Adithya Kini K., Sanil V.K. (2021). Experimental investigation of mechanical sustainability and acoustic performance of fly ash cenosphere/epoxy polymer composites. J. King Saud. Univ. Eng. Sci..

[37] Gobetti A., Cornacchia G., Ramorino G., Riboldi A., Depero L.E. (2021). EAF slag as alternative filler for epoxy screeds, an example of green reuse. Sustain. Mater. Technol..

[38] Dębska B., Lichołai L., Silva G.J.B. (2020). Effects of waste glass as aggregate on the properties of resin composites. Constr. Build. Mater..

[39] Revelo C.F., Correa M., Aguilar C., Colorado H.A. (2021). Composite materials made of waste tires and polyurethane resin: A case study of flexible tiles successfully applied in industry. Case Stud. Constr. Mater..

[40] Olcay H., Kocak E.D. (2021). Rice plant waste reinforced polyurethane composites for use as the acoustic absorption material. Appl. Acoust..

[41] Kuźnia M., Magiera A., Pielichowska K., Ziąbka M., Benko A., Szatkowski P., Jerzak W. (2019). Fluidized bed combustion fly ash as filler in composite polyurethane materials. Waste Manag..

[42] (2003). Paints and Varnishes—Determination of Viscosity Using Rotary Viscometers—Part 2: Disc or Ball Viscometer Operated at a Specified Speed.

[43] (2003). Plastics and Ebonite—Determination of Indentation Hardness by Means of a Durometer (Shore Hardness).

[44] (2019). Plastics—Determination of Tensile Properties—Part 1: General Principles.

[45] (2012). Plastics—Determination of Tensile Properties—Part 2: Test Conditions for Moulding and Extrusion, Plastics.

[46] (2016). Paints and Varnishes—Pull-Off Test for Adhesion.

[47] (2011). Paints and Varnishes—Rapid-Deformation (Impact Resistance) Tests—Part 1: Falling-Weight Test, Large-Area Indenter.

[48] (2019). Plastics—Determination of Flexural Properties.

[49] CEN (2014). EN 13892-3—Methods of Test for Screed Materials—Part 3: Determination of Wear Resistance—Böhme.

[50] Memon H., De Focatiis D.S.A., Choi K.S., Hou X. (2021). Durability enhancement of low ice adhesion polymeric coatings. Prog. Org. Coat..

[51] Krzywiński K., Sadowski Ł., Stefaniuk D., Obrosov A., Weiß S. (2021). Engineering and manufacturing technology of green epoxy resin coatings modified with recycled fine aggregates. Int. J. Precis. Eng. Manuf. Green Technol..

[52] Awaja F., Gilbert M., Kelly G., Fox B., Pigram P.J. (2009). Adhesion in polymer science. Prog. Polym. Sci..

[53] Momber A.W., Irmer M., Marquardt T. (2020). Effects of polymer hardness on the abrasive wear resistance of thick organic offshore coatings. Prog. Org. Coat..

[54] Malaki M., Hashemzadeh Y., Tehrani A.F. (2018). Abrasion resistance of acrylic polyurethane coatings reinforced by nano-silica. Prog. Org. Coat..

[55] Barbakadze K., Brostow W., Datashvili T., Hnatchuk N., Lekishvili N. (2018). Antibiocorrosive epoxy-based coatings with low friction and high scratch resistance. Wear.

[56] Yeasmin F., Mallik A.K., Chisty A.H., Robel F.N., Shahruzzaman M., Haque P., Rahman M.M., Hano N., Takafuji M., Ihara H. (2021). Remarkable enhancement of thermal stability of epoxy resin through the incorporation of mesoporous silica micro-filler. Heliyon.

[57] da Silva L.R.R., Avelino F., Diogenes O.B.F., Sales V.O.F., da Silva K.T., Araujo W.S., Mazzeto S.E., Lomonaco D. (2020). Development of BPA-free anticorrosive epoxy coatings from agroindustrial waste. Prog. Org. Coat..

[58] Sabu T., Sinturel C., Raju T. (2014). Micro and Nanostructured Epoxy/Rubber Blends. Properties of Materials.

